# Supplementary material for: Gestational weight gain in sub-Saharan Africa: Estimation based on pseudo-cohort design
Source: PLoS One. 2021 May 26;16(5):e0252247. doi: 10.1371/journal.pone.0252247 (PMC8153429; doi:10.1371/journal.pone.0252247)
Supplement: S1 File — (DOCX) [file pone.0252247.s001.docx]

**Control data for assessing trend in gestational weight gain**

Data of 89,444 women from 11 national surveys

Data of 468,717 women from 33 national surveys conducted between 2010 and 2020 was available

The data of 240,450 women (including the entire recent DHS conducted in Angola, Zambia and Mozambique) excluded because no bodyweight data was taken

Data of 228,267 women from 30 national surveys

6,312 women had no maternal weight data for various reasons

Data of 221,955 women from 30 national surveys

111,473 non-pregnant women not eligible for the analysis

Data of 110,482 women (88,602 non-pregnant and 21,822 pregnant women) from 30 national surveys conducted between 2010 and 2020 analysed

**Data for estimating mean gestational weight gain**

Data of 114,321 women from 13 national surveys conducted between 1997 and 2003 was available

Data of 86,626 women from 11 national surveys

Data of 50,603 women (40,072 non-pregnant and 9,055 pregnant women) from 11 national surveys conducted between 1997-2003 analysed

The data of 24,872 women (including the entire DHS conducted in Tanzania and South Africa) excluded because no bodyweight data was taken

2,823 women had no maternal weight data for various reasons

36,023 non-pregnant women not eligible for the analysis

**S1 File. Flow chart of the study.**
